# An Interface for Biomedical Big Data Processing on the Tianhe-2 Supercomputer

**DOI:** 10.3390/molecules22122116

**Published:** 2017-12-01

**Authors:** Xi Yang, Chengkun Wu, Kai Lu, Lin Fang, Yong Zhang, Shengkang Li, Guixin Guo, YunFei Du

**Affiliations:** 1 College of Computer, National University of Defense Technology, Changsha 410073, China; yangxi1016@nudt.edu.cn (X.Y.); kailu@nudt.edu.cn (K.L.); 2 Beijing Genomics Institute (BGI) Shenzhen, Shenzhen 518083, China; fangl@genomics.cn (L.F.); zhangyong2@genomics.cn (Y.Z.); lishengkang@genomics.cn (S.L.); 3 National Supercomputing Center of Guangzhou, Guangzhou 510006, China; guixin.guo@nscc-gz.cn; 4 School of Data and Computer Science, Sun Yat-Sen University, Guangzhou 510000, China; duyunfei@mail.sysu.edu.cn

**Keywords:** big data, Tianhe-2, Hadoop, Spark, genomics big data

## Abstract

Big data, cloud computing, and high-performance computing (HPC) are at the verge of convergence. Cloud computing is already playing an active part in big data processing with the help of big data frameworks like Hadoop and Spark. The recent upsurge of high-performance computing in China provides extra possibilities and capacity to address the challenges associated with big data. In this paper, we propose Orion—a big data interface on the Tianhe-2 supercomputer—to enable big data applications to run on Tianhe-2 via a single command or a shell script. Orion supports multiple users, and each user can launch multiple tasks. It minimizes the effort needed to initiate big data applications on the Tianhe-2 supercomputer via automated configuration. Orion follows the “allocate-when-needed” paradigm, and it avoids the idle occupation of computational resources. We tested the utility and performance of Orion using a big genomic dataset and achieved a satisfactory performance on Tianhe-2 with very few modifications to existing applications that were implemented in Hadoop/Spark. In summary, Orion provides a practical and economical interface for big data processing on Tianhe-2.

## 1. Introduction

In recent years, the so-called “big data” trend has become increasingly prominent due to the proliferation of large scientific instruments such as particle colliders and astronomical telescopes, as well as high-throughput analysis devices like Next-Generation sequencers and pervasive sensors. Many scientific discoveries and insights are now driven by massive datasets, and this is considered as the fourth paradigm of science besides experiments, theories, and computational methods [1]. The volume, velocity, and variety of data generation comprise the major challenges of big data. Biomedical studies emerge as a perfect example. For instance, the European Bioinformatics Institute (EBI, one of the largest biological data holders) stores over 20 petabytes of data, which recently has tended to double every year [2]. The heterogeneity of the data sources, including genomics, proteomics, metabolomics, microarray data, literature, etc., makes it even more complex. 

Timely analysis of those huge datasets is usually of great importance for scientific discoveries, economic decisions, and other activities. To address this challenge, advanced computing technologies are essential. 

The first solution is to utilize open source frameworks that were designed to run on commodity-level servers, clusters, computing grids, or cloud environments. 

Hadoop, following the MapReduce model, is one of the most representative programming frameworks for big data processing [3]. It is an open source Java-based programming framework that supports the processing and storage of extremely large data sets in a distributed computing or cloud computing environment. The framework makes it possible to run big data applications on systems constructed by connecting many commodity nodes. It specifically employs a number of mechanisms to enable applications to continue operating even in case of node failures, including the Hadoop Distributed File System (HDFS) [4]. It was designed to run on distributed systems, which are commonly built as a cluster of commodity hardware, which is relatively cheap compared to supercomputers built using high-end elements. Three major components of Hadoop include a distributed filesystem, a processing module, and a job manager. The HDFS manages distributed storage, and the processing core adopts the MapReduce paradigm. The MapReduce model “maps” the input data into subgroups (by grouping and sorting) and applies multiple maps functions in parallel on each subgroup of data. The “reduce” operation produces an output file for each reduce task. The MapReduce module hides all details of parallel processing, data transfers and recovery from errors. One disadvantage is that many applications exhibit computational and data access patterns that cannot be expressed as a MapReduce model. Moreover, Hadoop writes all intermediate data to disk, which involves a lot of slow IO operations.

The Hadoop framework is widely used in molecular research. For instance, since traditional methods are incapable of dealing with the large-scale data of high-throughput sequencing data and the DNA MSA (Multiple sequence alignment) issue, Zou, Q. [5,6] addressed the problem by using the Hadoop platform to exploit parallelism. Traditionally, chemists need to upload small molecule files and collect the virtual screening results manually, which is a laborious and tedious procedure. To alleviate this situation, Zhao, J. [7] developed a Hadoop-based application for large-scale data storage, which uses HDFS to store and manage small molecule files and docking results. By using the Hadoop programming framework for parallel docking, result files was preprocessed and the automation of the virtual screening molecular docking was achieved. Zhang, Y. [8] proposed a novel storage solution based on Hadoop, introducing HBase as a distributed database to maintain the properties of massive molecules and docking results. In addition, HDFS was also utilized as a molecule source files storage system. Niu, J. Ellingson [9] created AutoDockCloud, which implemented the MapReduce paradigm for distributed computing by using the Hadoop framework, demonstrating a speed-up of 450× on a commercial cloud service.

To further improve the data processing efficiency and the capability to address more complex tasks, the in-memory big data framework Spark has become increasingly popular [10,11]. Spark addresses several aspects of performance issues to accelerate big data processing. Firstly, it maximizes the utilization of the main memory of each node to avoid repeated IO requests for intermediate results; secondly, it employs a directed acyclic graph (DAG) mechanism for task scheduling, which is more efficient and flexible. Spark has attracted a great deal of attention, as it is more efficient than Hadoop (up to 100× speedup) and it provides support for Java, Python, Scala and R. It also provides a stack of libraries including SQL, MLlib for machine learning, GraphX for graph processing, and Spark Streaming for stream processing.

Numerous big data applications were developed using Hadoop and Spark [12,13]. To run Hadoop and Spark applications, you need a Hadoop/Spark cluster, whose scale can be from a few nodes to thousands. Big companies like Baidu, Tencent, and Alibaba usually maintains several dedicated large-scale clusters for such purposes. However, not everyone can have their own cluster. Cloud computing could be an alternative choice. Mainstream cloud computing services like Alibaba Cloud and Tencent Cloud do provide interfaces that can help you set up temporary Hadoop/Spark clusters for big data processing. Nevertheless, to use such services, you need to have a data “highway” to transfer your big data onto the cloud.

High-performance computing (HPC) like Tianhe-2 [14,15,16] represents high-end computing infrastructures that have traditionally been used to solve compute-intensive scientific and engineering problems. Although the computing power, stability, scalability, and storage capacity of HPC systems are enormous, they are usually expensive to build and deploy. In addition, profound experience in parallel programming (e.g., OpenMP, MPI) and knowledge about the system architecture are required in order to harness the power of HPC systems. Consequently, only a small number of engineers can utilize HPC to handle their big data problems. 

Although the software stack on Tianhe-2 is designed and optimized for compute-intensive tasks [17,18,19], its high-performance architecture does provide the possibility and capability of efficient big data processing. The motivation of this paper is to implement an easy-to-use interface on Tianhe-2 for Hadoop/Spark applications in a dynamic and scalable way. This is a non-trivial task for the following two reasons: firstly, the resource requirements and configuration settings vary for different users and different applications; for convenience, users might want to have full control over the configuration process. However, it is infeasible (or very costly) for the supercomputing center to perform a new configuration from scratch every time, and if given too much control, users might unintentionally (or maliciously) breach the security protocols; secondly, users are charged based on the amount of resources they occupy, so they would like to release spare resources whenever possible. That would require changes in the configuration, and it could be a tedious process to perform different configurations over and over again. Frequently, users might want to run existing big data applications built for cloud computing on the Tianhe-2 supercomputer without major modifications to the program. In addition, users often want to develop new applications in their familiar framework (Hadoop/Spark) that can run readily on Tianhe-2. 

In this paper, we first emphasize the need to enhance big data support on modern supercomputers, and we analyze the architecture of Tianhe-2 for a practicable and efficient solution. We propose Orion, which takes requests for big data processing, allocates the desired amount of resources, and automatically sets up and configures a recyclable Hadoop/Spark cluster using the HPC system software stack. The benefits are two-fold: firstly, developers avoid re-inventing the wheel by utilizing existing Hadoop/Spark applications; secondly, the Tianhe-2 software stack requires no major changes. We tested Orion using genomics big data processing as a case study, which comes from one of the major users of Tianhe-2: the Beijing Genomics Institute (BGI). The main contributions of this paper can be listed as follows:

1. We analyze the need for and possibility of convenient big data support on modern supercomputers
2. We propose Orion, which is an easy-to-use interface for users who wants to run Hadoop/Spark applications on the Tianhe-2 supercomputer
3. We test Orion on Tianhe-2 against an in-house Hadoop cluster using the genomics big data example and testify the utility of Orion

## 2. Results and Discussion

### 2.1. Automated Deployment of Big Data Applications

We developed the Orion big data interface to facilitate big data applications on Tianhe-2. In order to more efficiently solve multiple tasks, one user can launch multiple Orion instances to maintain several big data analytics “clusters” (composed of Tianhe-2 compute nodes) at the same time, so that each Orion instance can deal with a dedicated task. The maximum number of Orion instances is limited by the number of available compute nodes in the Tianhe-2 system.

**Figure 1:**
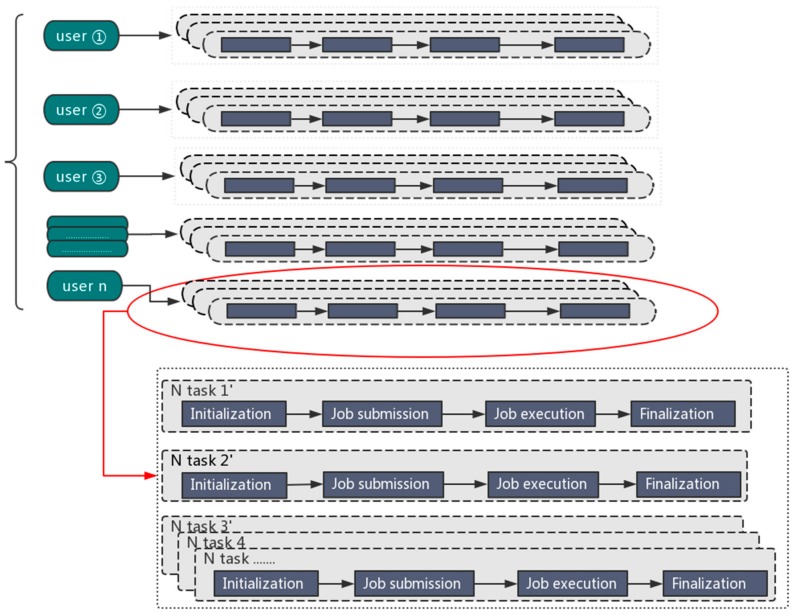
The parallel processing mode for big data analytics.

The parallel processing mode is illustrated in Figure 1.

### 2.2. Testing Orion: Genomics Big Data as a Case Study

In recent years, a deluge of genomic sequence data has been observed due to the constantly decreasing cost of sequencing. We are now entering the precision medicine era, in which medical discoveries will largely depend on the processing and analysis of large genomic data sets. In this paper, we collaborated with one major player in the field of genomics—the Beijing Genomics Institute (BGI)-Shenzhen—to test the utility of Orion in genomics big data processing. 

The key question is, can they readily run their existing applications on Tianhe-2? To answer this question, we tested the four major components that are essential for a genomics analysis pipeline, SOAPGaea, which is their primary analysis software (using the Hadoop framework). Those four components include FASTQ filtering, read alignment, duplication removal, and quality control. Four components were tested on a sample dataset of 300 GB (in BAM format). We carried out the test using the following three different scenarios. Details of the three scenarios are listed in Table 1. Note that in all three scenarios, some RAM must be reserved for system needs. 

The performance of each component under three different scenarios is listed in Table 2. Overall, we can see a major performance boost in Orion-1 and Orion-2 compared to the BGI setting (the shortest processing time is presented in bold and italic format). In Orion-1, although the number of running cores was fewer (6 cores, 10 cores in BGI), the amount of available RAM was far more than that of BGI (8 GB compared to 3 GB). In Orion-2, the number of running cores on each node was greater than that of BGI. Note that the CPUs utilized in BGI and Tianhe-2 are of different specifications, and so it is not a completely fair comparison. Without loss of generality, the performance comparison here is mainly to demonstrate that by using Orion, users can easily run their big data application on Tianhe-2 and they can gain better performance if using the same amount of compute nodes (as Tianhe-2 nodes have more powerful CPUs and more RAM). The cost of leasing Tianhe-2 nodes is quite low (about 2.4 RMB per node-hour). In addition, using Orion to run Hadoop applications is as simple as typing a command. Therefore, it is completely feasible to consider Tianhe-2 as a solution for BGI’s genomics big data analytics. 

In Table 2, we can also observe that the performance boost in Orion-1 and Orion-2 is actually not linear. This is because different components in SOAPGaea have different computing patterns. Some are sensitive to the amount of RAM, while others are sensitive to the number of cores.

The above tests demonstrate how BGI’s Hadoop applications can run easily on Tianhe-2 with the help of Orion. Below, we would like to demonstrate how it can be applied to BGI’s Spark applications. In Table 3, we tested Orion on one specific module named GaeaDuplicate Spark, which can be decomposed into three steps: read in → compute → write out. This module spends a lot of time in I/O. The input data is a 300 GB dataset in BAM format. We tested this module with three scenarios as well. 

Note that in Orion-A and Orion-B we only allowed the cores to use up to 70 percent of the node RAM to ensure node stability. The testing results are listed in Table 4.

As we can see, as the number of available nodes increases, the computation time is vastly reduced. The time needed for I/O constructs the barrier of further reducing the processing time. Here, the GaeaDuplicate module will be integrated into a running pipeline; that is, there would be one I/O operation in, many computation steps, and one I/O operation out. Therefore, the percentage of I/O time will be reduced, and the overall processing time can be greatly decreased if we introduce more compute nodes when launching Orion. 

In all, we have demonstrated that Orion can facilitate existing big data applications without requiring the users to modify their programs, while also providing a performance boost. The performance boost can be attributed to multiple reasons. Firstly, Tianhe-2 is extremely powerful as a whole system, and each node is also more powerful compared to commodity ones; secondly, the amount of RAM available on Tianhe-2 is more abundant, which fits well for big data applications. There are aspects where the performance can be further improved, like utilizing RDMA (Remote Direct Memory Access) [16]. However, that is beyond the scope of this paper, which focuses on providing an easy-to-use interface for big data users to run their applications readily on Tianhe-2. 

Currently, there are many molecular big data applications available or under development, using standard frameworks like Hadoop and Spark. To take full advantage of current efforts on cloud, we aim not to re-invent the wheel on the HPC platform but to develop an interface support at the platform level. In this manner, it is possible to boost current cloud applications by fully utilizing the computational and data access performance of HPC systems. 

### 2.3. Portability of the Interface

Besides Tianhe-2, a number of Tianhe supercomputing systems have been deployed all over China in recent years, hosted by national or local supercomputing centers in different areas of the country. For instance, Tianhe-1 and Tianhe-1A [14] are hosted by the National Supercomputing Center of Changsha and Tianjin, respectively. 

Different Tianhe HPC systems have different specific hardware architectures. However, the software environments are similar in terms of the job management system and the operating system, and it is relatively straight-forward to deploy Orion on different Tianhe HPC systems. Several recently delivered Tianhe HPC systems are already pre-configured with the Orion interface, all of which went through many iterations of functional and pressure tests. Consequently, users only need to modify some configuration parameters (like paths, partition names) to use Orion.

## 3. Materials and Methods

Orion is implemented via Shell scripts. It employs the scheduling system of Tianhe-2 to allocate required computing resources and utilizes the parallel filesystem of Tianhe-2 directly rather than HDFS.

**Figure 2:**
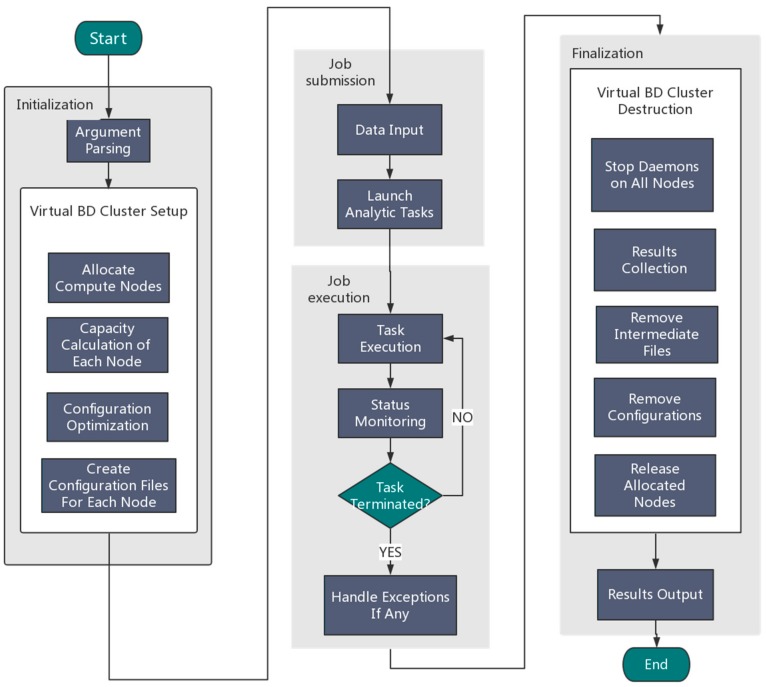
An overview of the Orion architecture for big data analytics.

An overview of the Orion implementation is depicted in Figure 2. Generally speaking, Orion involves the following four components:

(1) Initialization

Users need to specify the framework type (Hadoop or Spark) and the desired number of nodes. Orion parses the user input and then automatically allocates resources by calling the Tianhe-2 scheduling system to configure a virtual Hadoop/Spark cluster using allocated compute nodes.

(2) Job submission

Orion can either read an analytic job from a user job Shell script or accept a user job interactively using the command line.

(3) Job execution

Orion executes the submitted job and monitors the status of the execution in real time. Once the task terminates unexpectedly, exception handling will be started.

(4) Finalization

Orion collects the generated results and stores them in the globally shared filesystem. Orion will then stop all compute daemons and release allocated compute nodes.

### 3.1. Features of Orion

In summary, Orion has the following features:

- It supports Hadoop and Spark configuration automatically; the user only needs to specify the framework type and the number of nodes.
- We use the original scheduling system of Tianhe-2 and do not use YARN for node management.
- We use the H2FS filesystem of Tianhe-2 instead of HDFS.
- We not only configure the framework work environment, but also enable related environments like NoSQL databases support (Redis, Mongo DB), OpenMP, and MPI.
- The Tianhe-2 monitoring subsystem can track the status of the temporarily created big data analytics cluster dynamically.
- The temporary data analytics cluster can be created and recycled any time the users wish, so that users will not be charged if their applications are not actually running.

### 3.2. Demonstration of Orion Usage (Tianhe-2 in the NSCC Guangzhou as an Example)

**Figure 3:**
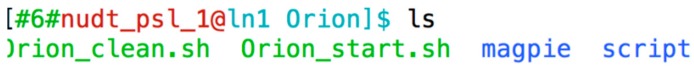
The initial interface of the installation directory.

**Figure 4:**
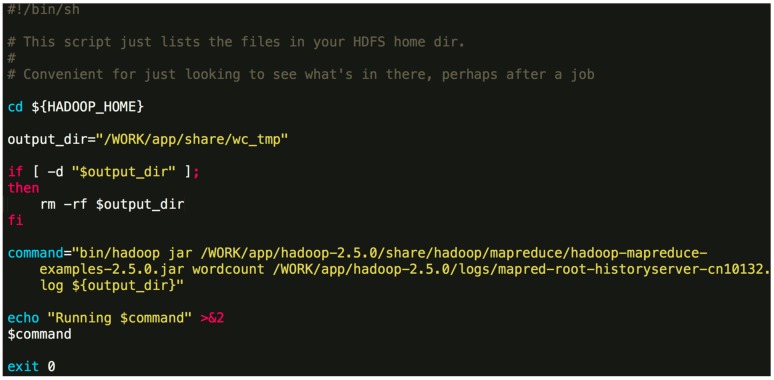
An example job script.

To use Orion and run big data applications (Hadoop/Spark) on Tianhe-2, the procedure is described in details as follows:

1. Log in to your user account on Tianhe-2 and you will be given a command line terminal. Then, you can see the following:
  - The installation directory
*/WORK/app/share/Orion*
  - The initial situation in this directory is as the Figure 3.
    Orion_start.sh is used for cluster configuration and start. Orion_clean.sh is the task run after the completion of the calculation or the release of resources upon an error, and cleans up the directory. Script directory is used to store task scripts.
2. Switch to the Orion installation directory and execute a command like:
*./Orion_start.sh -t hadoop -p work -N 6 -s ‘pwd’/script/wordcount.sh*
  Here, the “-t” option can either be “hadoop” or “spark”, which specifies the big data framework you are using; the “-p” option specifies the resource pool on Tianhe-2 you want to use; the “-N” option specifies the number of compute nodes to use; the “-s” option specifies the job Shell script to be executed. The first three options are obligatory. The last one is optional. An example job script “wordcount.sh” is given in Figure 4.
  The above command will invoke the initialization process and wait for the resources to be allocated. If the required resources cannot be allocated after a long time, Orion will prompt the user to select a different resource pool or to use less nodes.
  $ {HADOOP_HOME} -points to the directory where Hadoop is installed. In general, the contents of the command can be modified. You can also specify a different output directory.
3. Once the task is submitted, it will be automatically queued to wait for resources. If the designated resource requirements cannot be fulfilled, Orion will give a message and you will need to switch to a partition with more idle resources or reduce the number of nodes.
4. If the application for resource is successful, Orion will continue to configure and launch a Hadoop/Spark cluster using the allocated nodes.
5. Orion executes the designated script and waits for the execution to finish. You can continue to use the configured cluster to run jobs interactively.
6. Use a command like the following to terminate the cluster and release occupied resources:
*./Orion_clean.sh -j 1362049*
  Here the “-j” option tells Orion to clean up the corresponding job, and the job ID is given in Step 2.

Tips:

Regarding the configuration file changes:

If you want to modify the configuration file, you can switch to the installation directory under the magpie/conf directory, and modify the corresponding file. The changes will take effect when you create a new virtual cluster. You can also manually log in to the master node of the existing cluster and restart YARN and JobHistoryServer as described above.

## 4. Conclusions 

In this paper, we proposed Orion, the big data analytics platform on Tianhe-2, which enables users to submit their current big data applications to Tianhe-2 without many changes and to gain a performance boost at the same time. As Orion manages all details about communicating with the supercomputer, users with little knowledge about Tianhe-2 can also develop new Hadoop/Spark applications that can run on Tianhe-2 via Orion. We tested Orion using genomics big data as a case study. Through Orion, BGI’s current applications can run on Tianhe-2 readily with a major performance boost, which can be attributed to the hardware advantages and the sufficient amount of computational resources of Tianhe-2. In summary, Orion provides an easy-to-use and scalable interface to users who want to carry out big data analytics on the Tianhe-2 supercomputer, and this interface is also available for other Tianhe HPC facilities. The use is straightforward, and the performance is satisfactory. 

For future work, we will introduce more optimization to further improve the big data performance on Tianhe-2. 

## Figures and Tables

**Table 1:** Hardware and software settings of the Hadoop test. BGI: Beijing Genomics Institute; HDFS: Hadoop Distributed File System.

Test Scenario Name	Hardware Setting	Analysis Setting
BGI	A Hadoop cluster with 18 nodes. Each node is equipped with 24 cores, 32 GB RAM. The storage uses HDFS, and the total capacity is 12 TB.	1 master + 17 slaves.Each core is allocated 3 GB RAM, a maximum of 10 cores were used on each node.
Orion-1	A Hadoop cluster initiated and maintained by Orion on Tianhe-2 with 18 nodes. Each node is equipped with 24 cores, 64 GB RAM. Use the Tianhe-2 parallel filesystem directly.	1 master + 17 slaves.Each core is allocated 8 GB of RAM, a maximum of 6 cores were used on each node.
Orion-2		1 master + 17 slaves.Each core is allocated 3 GB of RAM, a maximum of 16 cores were used on each node.

**Table 2:** Performance comparison of four components of SOAPGaea.

SOAPGaea Components	BGI	Orion-1	Orion-2
FASTQ Filtering	24 m 43 s	12 m 50 s	10 m 23 s
Read Alignment	1 h 35 m 56 s	48 m 48 s	49 m 49 s
Duplication Removal	28 m 21s	15 m 38 s	9 m 43 s
Quality Control	1 h 30 m 2 s	1 h 39 m	46 m 38 s
Total processing time	3 h 59 m 2 s	2 h 56 m 16 s	1 h 56 m 33 s

**Table 3:** Hardware and software settings of the Spark test.

Test Scenario Name	Hardware Setting	Analysis Setting
BGI	A Spark cluster with 18 nodes. Each node is equipped with 24 cores, 32 GB RAM. The storage uses HDFS and the total capacity is 12 TB.	1 master + 17 slaves. Each core is allocated 3 GB RAM, a maximum of 10 cores were used on each node.
Orion-A	A Spark cluster initiated and maintained by Orion on Tianhe-2 with 100 nodes. Each node is equipped with 24 cores, 64 GB RAM. Uses the Tianhe-2 parallel filesystem directly.	1 master + 99 slaves. 24 cores, a maximum total of 44 GB RAM cores were used on each node.
Orion-B	A Spark cluster initiated and maintained by Orion on Tianhe-2 with 250 nodes. Each node is equipped with 24 cores, 64 GB RAM. Uses the Tianhe-2 parallel filesystem directly.	1 master + 249 slaves. 24 cores, a maximum total of 44 GB RAM cores were used on each node.

**Table 4:** Performance decomposition for GaeaDuplicate Spark in different settings.

GaeaDuplicate_Spark	Read In	Compute	Write Out	Total
BGI	17 m	1.1 h	40 m	2 h
Orion-A	25 m	14 m	40 m	1.3 h
Orion-B	32 m	6 m	25 m	1.1 h
